# The Impact of Microgravity on Immunological States

**DOI:** 10.4049/immunohorizons.2200063

**Published:** 2023-10-19

**Authors:** Janelle Hicks, Makaila Olson, Carol Mitchell, Cassandra M. Juran, Amber M. Paul

**Affiliations:** * Department of Human Factors and Behavioral Neurobiology, Embry-Riddle Aeronautical University, Daytona Beach, FL; † NASA Ames Research Center, Space Biosciences Division, Moffett Field, CA; ‡ Blue Marble Space Institute of Science, Seattle, WA

## Abstract

As we explore other planetary bodies, astronauts will face unique environmental and physiological challenges. The human immune system has evolved under Earth’s gravitational force. Consequently, in the microgravity environment of space, immune function is altered. This can pose problematic consequences for astronauts on deep space missions where medical intervention will be limited. Studying the unique environment of microgravity has its challenges, yet current research has uncovered immunological states that are probable during exploration missions. As microgravity-induced immune states are uncovered, novel countermeasure developments and personalized mitigation programs can be designed to improve astronaut health. This can also benefit immune-related monitoring programs for disorders on Earth. This is a comprehensive review, including gaps in knowledge, of simulated and spaceflight microgravity studies in human and rodent models.

## Introduction

The immune system defends against various external and internal challenges and is integrated with virtually every other physiological system. Thus, understanding immunity in response to the unique environment of space is paramount to support future missions. Both lymphatic and circulatory systems, which in part house the human immune system, develop and function in the presence of Earth’s gravitational force (1 *g*, Earth’s standard) (1). As part of immune cell function, mechanotransduction is the process of how cells sense and convert mechanical gravitational force signals into biochemical responses (2). Microgravity is common terminology for the spaceflight environment where the acceleration of gravity experienced by an object is greatly reduced and is defined as 10^−3^ to 10^−6^ *g* to Earth’s standard (3). In this environment, mechanotransduction and fluid dynamics are altered. Muscular atrophy, vascular remodeling from increased venous pressure-endothelial damage, and deconditioning loss of hydrostatic lymphatic and interstitial pressure are all consequences of exposure to the microgravity environment in spaceflight (4). In addition, redistribution of fluids to cavities, such as the thorax and head, which would otherwise be localized to lower extremities on Earth, can impact immune mobilization and activity (2, 5).

Human and rodent immune data in the microgravity environment are gathered through ground-based analogs, the International Space Station (ISS), or short-term commercial spaceflights (6). Retrospective immune data collected during the Apollo era and shuttle missions also offer valuable information on this unique environment (7, 8). However, data describing the effects of deep space and long-duration missions on immune and other physiological performances are limited, and acquisition of such data is essential for planning future missions. Furthermore, ground studies using simulated microgravity models in controlled environments can produce comparable results to spaceflight. However, a caveat is the physical properties of spaceflight versus simulated microgravity, which are difficult to effectively emulate. Free-fall unloading in the microgravity environment is experienced on board the ISS, which orbits the Earth at a precise speed and altitude (3). Simulated microgravity differs from spaceflight microgravity. For instance, in cell culture, simulated microgravity either randomizes or produces omnidirectional gravity acceleration vectors, which is in contrast with constant free fall that is experienced in spaceflight microgravity. Further, in animal models, reduced weight bearing and redistribution of fluids are a function of simulated microgravity. However, both simulated modeling and spaceflight microgravity can generate comparable biological consequences. The benefit of simulated microgravity modeling is that it isolates out confounding risk factors, such as cosmic radiation, psychological stress–induced social isolation, and hypergravity environments experienced during the launch and landing phases of spaceflight, which all produce distinct responses.

Understanding the consequences of reduced gravity on immune performance during and after spaceflight are critical for exploratory missions where gravitational force will change relative to the nearest celestial body, i.e., the Moon or Mars. This change in gravity-driven loading will certainly be experienced by humans on these missions resulting in biological responses. Therefore, readaptation and recovery are important not only for terrestrial return but for lunar (0.166 g to Earth’s standard) and Martian (0.376 g to Earth’s standard) missions as well, where gravitational forces experienced by organisms are less than Earth’s (9, 10). Due to the complex and diverse nature of the immune response, a thorough analysis of longitudinal immune states in response to differential gravity environments are required for improved astronaut health-monitoring programs and countermeasure implementation.

Immunological states that develop as a consequence of exposure to the microgravity environment display similarities to an aged immune system. In line with this, accelerated cellular aging has also been described after exposure to the spaceflight environment (11, 12). Aged immunological states include features of immune exhaustion, senescence, and inflammaging. By definition, immune exhaustion is a result of continuous leukocyte stimulation resulting in reduced proliferation and enhanced inhibitory coreceptor programs (13–16). Immune senescence also engages inhibitory programs on leukocytes, with reduced proliferation, and is associated with mitochondrial dysfunction (15, 17–19). Finally, inflammaging is a process of persistent, mild inflammation that is typically associated with aged immune systems (20, 21). Indeed, all of these immunological states or their features have been described in multiple spaceflight and ground microgravity studies.

In brief, this perspective surveys current knowledge on immune responses to simulated and spaceflight microgravity and postflight readaptation to gravity, and consolidates different platform results into a comprehensive timeline of key immunological states in the microgravity environment.

## Simulated Microgravity Experiments

**FIGURE 1.**
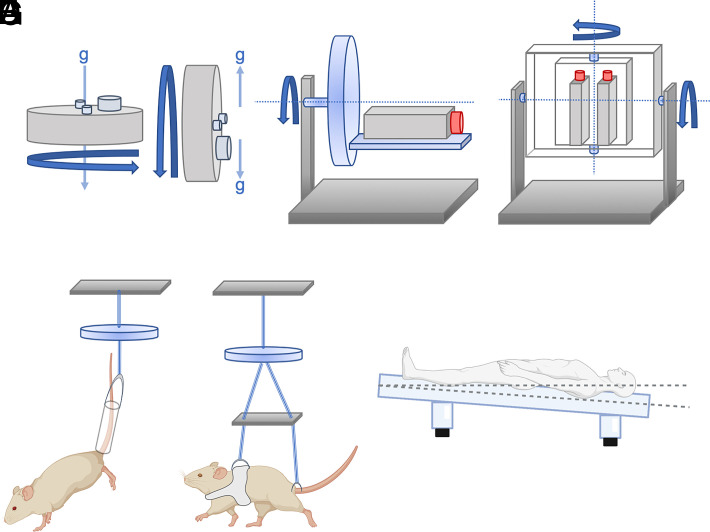
Representative image of common cell culture and animal microgravity analogs. RCCS-rotating wall vessel system dynamic control along the vertical axis (**A**) and simulated microgravity along the horizontal axis (**B**). 2D rotation along the horizontal axis, clinostat (**C**), and 3D rotation along the horizontal and vertical axis, clinostat (constant speed/direction) or random positioning machine (random speed/direction) multiaxis rotation (**D**). Hindlimb unloading (15–30° to the horizontal) model (**E**) and PWS (**F**) in rodents to model fluid shifting and reduced weight bearing. (**G**) Head down tilt bed rest (6° to the horizontal) in humans models fluid distribution shifting that is similarly experienced in spaceflight. Created with BioRender.com.

Simulated microgravity experiments allow researchers to conduct authentic, ground-based immune studies that are challenging to study in space. Common in-flight limitations include crew time, cost, and the dynamic nature of fluids in microgravity that are difficult to manipulate. Microgravity analogs include the rotating cell culture system (RCCS), also known as the rotating wall vessel platform, and the two-dimensional (2D)/three-dimensional (3D) clinostats or random positioning machines for cell culture studies (22). In animal studies, hindlimb unloading (HU; rodent) (23), partial weight suspension (PWS; rodent), and head down tilt bed rest (HDTBR; humans) (24) are commonly used microgravity analogs (Fig. 1). Other known but less commonly studied simulated ground analogs include diamagnetic (magnetic) levitation (25), ballistic rocket free fall (26–28), parabolic flight (26, 29), drop towers (10), and wet/dry immersion (24, 30), which are promising immune modeling platforms that can expand future research. Ground-based simulated microgravity platforms isolate out confounding variables, such as ionizing radiation, psychological stress responding to isolation, motion sickness from inner ear fluid shifts, etc., which are all experienced during spaceflight. However, ground-based analog platforms come with limitations compared with microgravity experienced in spaceflight, such as the physical nature of spaceflight microgravity (gravity loading force is reduced) versus simulated microgravity (gravity force exists as either omnidirectional, randomized, or reduced weight bearing). Yet, without these analogs, the current breadth of research possible to assess immunological consequences in this unique environment would be severely limited.

### Simulated microgravity experiments in vitro

Most in vitro immune cell culture studies are conducted over the course of a few hours to days and highlight the prompt immune response to simulated microgravity. Research on innate cell types, including basophils and eosinophils in simulated microgravity, is limited. However, one study identified reduced bone marrow–derived mast cell degranulation and increased apoptosis in RCCS-simulated microgravity (15 rpm for 24, 48, and 168 h) (31). This is indicative of impaired early- and late-phase mast cell activation. In another study, neutrophil activation via reactive oxygen species (ROS) and myeloperoxidase expression increased after RCCS (20 rpm for 12 h) in human WBCs, suggesting mild inflammatory responses were engaged in simulated microgravity. This study further demonstrated that the inflammatory response was partially mitigated with antioxidant (*N*-acetyl cysteine) treatment (32), suggesting antioxidant treatment mitigates ROS-driven inflammatory cascades and may be a valuable countermeasure to consider for future studies.

Circulating monocytes and tissue-specific macrophages have been extensively studied in the microgravity environment (33), revealing cell-subtype–, protocol-, and analog-specific outcomes. Interestingly, reduced ROS was observed in a zymosan-stimulated alveolar macrophage cell line, NR8383, exposed to 2D clinostat (60 rpm for 20 min) simulated microgravity and during microgravity periods of parabolic flight, suggesting ROS may be depressed in the absence of gravity (34). In addition, murine macrophage cell line RAW 264.7 differentiated M0, M1, and M2 cells cultured on Cytodex 1 microcarrier beads were exposed to RCCS-simulated microgravity (14 rpm) resulting in reduced TNF-α across all polarized cell subtypes, with increased IL-12, IL-10, and vascular endothelial growth factor levels at 72 h postsimulation, indicating cell-specific differential cytokine programs are generated in response to simulated microgravity (35). Interestingly, M1 (proinflammatory) polarized macrophages displayed increased IL-6 that was not detected in M0 and M2 populations (35), further supporting distinct immune profiles in response to simulated microgravity. Various other macrophage subtypes (primary and cell lines) in simulated microgravity display either elevated (36, 37) or decreased (38, 39) proinflammatory IL-1, IL-6, IL-8, MCP-1, and TNF-α cytokine expression levels depending on experimental platform, protocol, and cell type, making interpretation further convoluted and inconclusive. Therefore, consolidation and integration of immune outcomes into a comprehensive map with experimental metadata may benefit future research queries (Supplemental Tables I, II).

Research on adaptive immune lymphocytes in the microgravity environment is rich. For one, primary human CD4^+^ T cells exposed to 2D clinorotation (60 rpm) for 5 and 60 min displayed reduced CD3 and ZAP70 abundance (5 min) and IL-2R (60 min), suggesting T cell signaling is impaired, resulting in reduced cell proliferation in microgravity (40). Another study identified reduced surface expression of IL-2R and CD69 and inhibited cell-cycle progression in human PBMCs stimulated with anti-CD3 and 2D clinorotation at 30 rpm up to 24 h, which indicated inhibition of cell proliferation is independent of TCR stimulation in the microgravity environment (41). To assess downstream T cell stimulation pathways of the TCR (CD3) signaling components, diacyl glycerol signaling transduction pathways in primary CD4^+^ human T cells after RCCS simulation (14 rpm, up to 90 min) was assessed, yet no downstream pathways were affected, suggesting simulated microgravity prevents T cell activation by modulating the T cell response and not the TCR signal itself (42). Pathways involved may include inhibitory signaling regulation and/or other undefined regulatory pathways. In line with this, lymphocyte proliferation was also reduced after 24 h of RCCS (10 rpm) microgravity simulation (43), collectively indicating suppressed lymphocyte function.

Cellular impacts of dysfunctional T cells were also described in one study that used RCCS (5 rpm for 18 h) and mass cytometry to measure human (21–55 y old) immune responses, which displayed suppressed CD4^+^, CD8^+^, and NK effector cell activity and enhanced T_regulatory_ responses (44). Another RCCS (10 rpm) study showed reduced human lymphocyte activity that was translated through suppressed IgM and IgG Ab production at 72 h and persisted up to 20 d with continuous simulated microgravity exposure (43), indicating depressed B cell or CD4 T cell activity. Additional studies using mice splenocytes cultured in RCCS (6 rpm for 48 h) in the presence or absence of PHA stimulation resulted in reduced IL-1β, IL-2, IL-3, TNF-α, and IFN-γ expression levels, along with reduced splenocyte proliferation (45). Therefore, multiple lymphocyte disparities are observed in the simulated microgravity environment, while further characterization of these disparities is ongoing.

To assess T cell engagement programs, unstimulated murine JAWS II dendritic cell (DC) line cells and mouse bone marrow–derived DCs were cultured up to 14 d to determine DC immunogenicity under simulated microgravity via RCCS (16 rpm) on microcarrier beads (46). The results showed increased p-STAT-5, p-ERK1/2, and p-mTOR signaling and upregulation of maturation markers MHC class I, MHC class II, CD80, and CD86. In addition, activation of Ag-specific CD4^+^ and CD8^+^ T cells was also shown up to 3 d in culture. However, between 4 and 14 d in culture (defined as long-term cell culture) RCCS-simulated DCs displayed reduced expression of maturation and T cell immunogenicity, suggesting biphasic and dynamic immune response kinetics are produced in the microgravity environment, which may be a function of immune adaptation. Similarly, JAWS II DC cells were cocultured with OT-II T cells (precultured for 24 h) in simulated RCCS at 14 rpm, and at an additional 24 h the capacity of T cell activation was accessed. OT-II T cells cocultured with JAWS II DCs and OVA peptide under RCCS-simulated microgravity produced elevated IL-2 activity compared with static controls, suggesting microgravity induced activation of T cells, and further indicates immune synapse communication is engaged in simulated microgravity. However, OT-II T cells cultured for 96 h in RCCS, followed by coculture addition of DC and OVA peptide for an additional 24 h, displayed significantly reduced IL-2 production, compared with static controls, suggesting T cell resistance occurs after longer duration of RCCS simulation. Further analysis revealed OT-II T cells produce elevated CTLA-4 expression at 120 h of RCCS simulation, compared with static controls, suggesting T cell exhaustion profiles are engaged in later stages of immune activation in the microgravity environment (47). Indeed, exhausted or immune senescent T cells produce elevated inhibitory coreceptors, including CTLA-4, which are also elevated in aged immune systems (13–15), further supporting immunological states associated with simulated microgravity cell culture platforms display elements of leukocyte immune exhaustion, senescence, and impaired proliferation. Furthermore, distinct cell- and exposure duration–specific inflammatory cytokine production is also produced in various phases of the immune response. Thus, future research should evaluate time-dependent, cell-specific responses to the microgravity environment to better identify collective immune networks involved.

### Simulated microgravity experiments in vivo: animal models

Animal models can evaluate integrative, multiphysiological system interactions and can enhance knowledge obtained from cell culture studies. Rodents can also be genetically modified or humanized to complement human immune research. Simulated microgravity studies using rodent animal models typically use two main methods, HU and PWS. HU involves the suspension of rodents by their hindlimbs/lower torso at an approximate 15–30° angle to cause fluid shifting toward the head and forelimb extremities, mimicking fluid shifts that are experienced in spaceflight (48). Alternatively, PWS results in a possible 10–80% reduced load on all four limbs compared with normal weight-bearing controls, with the goal of simulating low gravity conditions, such as lunar and Martian gravity (49).

Various simulated rodent microgravity studies have been conducted typically for an experimental duration of days to weeks. For example, 6- to 8-wk-old female *ICR* mice exposed to either HU or PWS (at 16% weight bearing) for 10 d displayed elevated WBCs, neutrophils, lymphocytes, monocytes, and eosinophil counts in blood at 24 h in HU, but not in PWS. These differences remained for WBC and neutrophil counts from day 2 until day 7 (50). Differences in neutrophil counts suggest fluid shifting–induced or lack of weight bearing–caused neutrophilia; however, more research is needed to determine the cause of neutrophil recruitment and the removal programs involved. The observation of elevated WBC and neutrophil counts is supported in a study of 16-wk-old C*57BL/6NJ* female mice exposed to HU. In this study, mice displayed a progressive increase in blood-circulating neutrophils at 14 and 30 d of HU. In this article, the number of lymphocytes remained constant, increasing the neutrophil-to-lymphocyte ratio (32), a marker for subclinical inflammation in metabolic and cardiovascular disorders (51, 52), and thus further supporting the contribution of elevated neutrophil mobilization in microgravity-induced pathology and highlighting the need for neutrophil migration studies as important avenues for future research in microgravity.

In another study, 5- to 6-wk-old female *ICR* mice exposed to HU for 4 d displayed increased blood circulating acute-phase LPS binding protein and soluble CD14 at 24 h of HU (53). Increased acute-phase reactant proteins in blood circulation may be caused by increased inflammation-induced disruption of the intestinal (gut) barrier and microbiome by the microgravity environment. Indeed, dysfunctional gut immunity may be critically involved in promoting mild inflammation described in spaceflight microgravity in humans (54) and rodents (55). For instance, 8- to 10-wk-old *C57BL/6* mice were exposed to HU for 14 d with dextran sodium sulfate (DSS)-induced colitis on day 7 of HU. DSS-treated HU mice experienced a 4-fold expansion of bacterial species and a 2-fold increase of neutrophil counts and IL-1β expression on day 14, compared with control mice (56). Another study indicated intestinal microbiota changes induced by microgravity altered epithelial cell integrity and increased mouse susceptibility to colitis. In this study, 8- to 10-wk-old *C57BL/6* mice were exposed to HU for 28 d, with DSS treatment on day 21. The results revealed an increased prevalence of *Clostridium* and reduced colonic goblet cell numbers in DSS-treated HU mice, exacerbating interaction between bacteria and host, causing dysbiosis (57). Similar outcomes may be experienced in spaceflight, because elevated *Clostridiales* and decreased *Lactobacillales* are observed in rodents (58).

Further evidence supportive of HU loading-altering gut microbiota and regulating immune recruitment was demonstrated in 6- to 8-wk-old male and female *C57BL/6* mice that underwent HU for 22 d and were challenged daily with *Citrobacter rodentium* on days 14–22 to induce diarrhea, colitis, and morbidity. A separate cohort of mice was fed a probiotic, VSL#3 (eight strains of highly concentrated probiotics) (59) for 22 d and challenged daily with *C. rodentium* on days 14–22. The results revealed increased host susceptibility to infection in HU-exposed mice, which was partially mitigated in the presence of probiotic treatment through restored gut dysbiosis and diversity of bacterial gut communities (60). Thus, future research should aim to develop and implement countermeasures, such as probiotics, into crewmember diets to enhance microbiome diversity and gut barrier integrity. Further considerations for enhancing nutritional value and reducing sterility of current food products on orbit may also promote healthy gut microbiome and immune system interaction.

Cross-talk between other physiological systems highly responsive to stressful environments, such as the neuroendocrine system, are also necessary to consider as immune modulators in the microgravity environment. Although many studies have profiled and characterized stress responses in microgravity environments, there is minimal knowledge on the threshold (hormone concentration), exposure duration (acute versus chronic), and immune cell-type-specific responses. For example, 5- to 6-wk-old male and female *ICR*, *BALB/c*, and *C3H/HeN* mice were exposed to HU for 10 d. On day 5, mice were challenged with *Pseudomonas aeruginosa* (systemic infection) or *Klebsiella pneumoniae* (respiratory infection), and bacterial clearance was measured. HU caused reduced clearance of bacteria by impairing peripheral blood granulocyte mobilization and elevated corticosterone levels (61). Although the effects of corticosterone on granulocytes in HU were not determined in this study, it is well known that stress hormones alter immune activity through multiple pathways, including inhibiting proinflammatory cytokines, shifting Th1 to Th2 lymphocyte phenotypes impacting cell-mediated immunity, impairing effector functions of macrophages and NK cells, and diminishing neutrophil and APC trafficking (62). Therefore, understanding complex interactions between stress (and sex) hormones on immune performance is an essential avenue for future microgravity and spaceflight research.

Aside from responding to microorganism challenges, effective neoplastic recognition strategies will also need to be in place to maintain homeostatic immunity on deep space exploration missions, where exposure to cancer-inducing radiation schemes will be experienced (63). Recently, a humanized mouse study showed acceptor mice that received human lymphocytes exposed to simulated RCCS microgravity (10 rpm for 12 h) experienced greater tumor burden than acceptor mice that received 1 *g* control human lymphocytes, suggesting simulated microgravity caused defective antitumor activity of lymphocytes. Furthermore, IL-2 and zoledronic acid (promoting NK and γδ T cell function) treatment partially rescued the antitumor effects through mechanisms that require further evaluation, and therefore may be an important countermeasure to consider for future deep space missions (64).

### Simulated microgravity experiments in vivo: human

The most commonly performed ground analog to study simulated microgravity in humans is HDTBR, whereby the participant lies on their back at a tilted downward angle (6° commonly used) parallel to the horizon, which mimics fluid shifting experienced in spaceflight and can alter immune states (24, 65). For example, males exposed to HDTBR for 5 d displayed reduced T lymphocyte populations and leukocyte adhesion molecule (CD62L) expression on granulocytes by day 3, which returned to baseline by day 5 and in recovery at day 4 post-HDTBR (66). Elevated soluble CD62L shedding is indicative of activated TLR-MYD88 and -IRAK4 signaling pathways, whereas reduced soluble CD62L levels suggest impaired microbial sensing and neutrophil function (67). Interestingly, increased soluble CD62L shedding was detected on day 3 in blood, whereas proinflammatory mediators IL-6 and IL-8 were no different compared with controls. These results indicate neutrophil activation is engaged early in simulated HDTBR, independent of typical inflammatory activation. Thus, the authors concluded neutrophil mobilization may be in response to mechanical shear stress because of fluid redistribution (68). These conclusions merge mechanotransduction receptor function of immune cells with pattern/pathogen recognition receptor immune activity, and thus indicating dual roles for mechanotransduction receptors in immune cells: to promote immune activity and to maintain cellular architecture in response to mechanical stress. For example, downregulation of mechanosensitive Piezo1 receptors in macrophages results in reduced inflammation and enhanced wound healing (69). Other notable mechanotransduction proteins include integrin receptors that are expressed in various immune cells (70), which perceive extracellular matrix mechanical stimuli and transmit signals to actin cytoskeletal proteins. These proteins then engage contractile or elastic properties in cells such as macrophages, neutrophils, and DCs undergoing migration and phagocytosis (71). Therefore, future research may focus on cell-specific expression patterns of mechanotransduction receptors and their inflammatory signaling pathways engaged in the microgravity environment, which may be important for countermeasure-targeted suppression of inflammation.

Another study assessed HDTBR in six male volunteers (aged 23–38 y old) for 120 d to determine peripheral blood immune and stress hormone profiles (72). Interestingly, β_2_ integrin expression on peripheral blood neutrophils doubled by day 65 and returned to near baseline during recovery (6 d post-HDTBR). Further, IL-6 was significantly elevated (32.66 ± 9.16 pg/ml) at day 65 compared with controls (8.44 ± 3.38 pg/ml) that returned to baseline levels in recovery (9.95 ± 2.74 pg/ml), collectively indicating elevated immune activity during HDTBR (72). Moreover, the CD4/CD8 ratio significantly decreased on days 65 and 105, whereas CD16^+^/CD56^+^ NK cells continuously increased, further adding to the complex dichotomy of the immune response in this unique environment (72). Diurnal night cortisol hormone production was also elevated (12.28 ± 2.32 nmol/l) by day 105, compared with baseline controls (3.91 ± 1.62 nmol/l) that persisted into recovery (day 6 post-HDTBR, 12.73 ± 3.07 nmol/l) (72), which may affect immune outcomes long term (62) and further highlights the necessity to explore integrative physiological cross-talk in future studies.

Aside from dysregulated inflammation, cell-mediated immunity has also been shown to be affected during and after exposure to simulated microgravity. One study by Kelsen et al. (73) exposed eight male patients (aged 26–38 y old) to 21 d of HDTBR. The results showed reduced blood Th1 cytokines IL-2, IFN-γ, and TNF-α at day 21, which returned to baseline at recovery (6 d post-HDTBR). Because Th1 cells/cytokines can activate phagocytic functions of macrophages and DCs and play an important role in directing cell-mediated immunity, deficiency in key Th1 cells/cytokine production in HDTBR indicates impaired immunity to pathogen or tumor challenge. These results further suggest T cell anergy or exhaustion profiles may occur during HDTBR.

To a large extent, all described HDTBR studies display rapid readaptation, and immune recovery is achieved by 1 wk, independent of the duration of simulated microgravity exposure. Conversely, noted biomarkers, including CD62L shedding and neuroendocrine cortisol persistence, which do not rapidly recover postexposure, may require extra attention for countermeasure intervention.

## Spaceflight Microgravity Experiments

Although abundant in the scientific literature, understanding the complex dynamics of the immune system in spaceflight microgravity is difficult. Gaps in knowledge include the extent of immune dysfunction as a measure of duration in space, key adaptive mechanisms involved in microgravity, the impact of microgravity on not just immune phenotypes but overall immune function, and recovery mechanisms within various gravitational environments. This perspective will discuss some studies conducted on board the ISS and shuttle missions aiming to forward research on these important topics.

**FIGURE 2.**
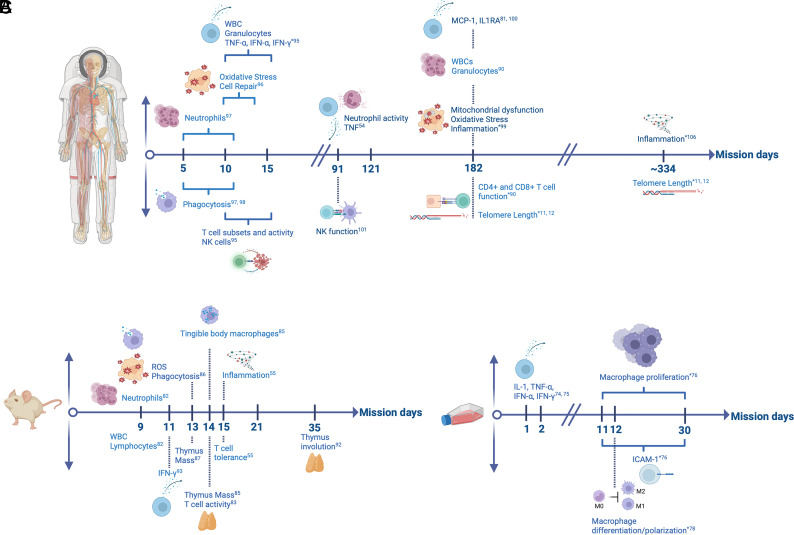
Representative summary of spaceflight microgravity studies (Refs. 11, 12, 54, 55, 74–76, 78, 81–83, 85–87, 90, 92, 93, 95–101, 106). (**A**) Human mission days are displayed, with postflight analysis results described. (**B**) Rodent model mission durations and postflight analysis results are displayed. (**C**) In-flight cell culture experimental results are presented, including mission day duration. Upward pointing arrows denote upregulation or increased activity/function; downward pointing arrows denote downregulation or reduced activity or function. Open circles indicate experimental or mission day start. * denotes in-flight collections with post-flight results described. Results of all studies are further described in Supplemental Tables I and II. Mission days are not to scale. Created with BioRender.com.

### Experiments in spaceflight microgravity: cell culture

Due to spaceflight constraints, conducting immune cell culture experiments on orbit that assess the effects of microgravity are limited. Still, experiments that have been performed provide researchers with essential information on functional consequences of immune cells in this unique environment. Generally, various bone marrow–derived macrophages, splenocytes, and lymph node cells cultured in space indicate cell-specific increased expression of IL-1, TNF-α, IFN-α, and IFN-γ cytokines compared with ground controls at multiple time points poststimulation, suggesting elevated immune activity in spaceflight (74, 75).

One study focused on metabolism and cytoskeletal alterations in primary human macrophages (76). Differentiated M1 (proinflammatory) macrophages were exposed to microgravity for 11 and 30 d on board the ISS in the CellBox-Primary Human Macrophages in Microgravity Environment payload. The results showed increased cellular area and number on board the ISS, indicating the spaceflight environment promoted macrophage proliferation, along with decreased ICAM-1, a leukocyte integrin recruitment adhesion molecule, expression on day 11 (76). Indeed, in situ macrophage proliferation is a feature of Th2 immunity and is a mechanism engaged to prevent infiltration of inflammatory cells into sites of activity by increasing localized effector cell macrophage numbers (77); therefore, in the spaceflight environment, localized inflammatory cascades may be dampened.

Characterization of other macrophage phenotypes, such as M2 (tolerogenic/anti-inflammatory) populations, is also necessary to understand the complexities of the immune response in the spaceflight microgravity environment. For one, overall reduced hematopoietic stem cell differentiation into M0 macrophage populations in spaceflight and simulated microgravity environments have been observed, along with reduced macrophage differentiation/polarization phenotypes (M1 and M2) and expression of ICAM-1 in flight (35, 76, 78, 79). These data support limited migratory behavior and activity of immune cells in microgravity, which can dampen overall immune performance. However, based on current knowledge, in situ migratory behavior analysis of leukocytes in the spaceflight microgravity environment has yet to be performed. The NASA Twins study identified in-flight M1 and postflight M2 predominance by single-cell RNA sequencing of lymphocyte-depleted blood on a 1-y mission (∼340 d), suggesting tolerogenic and repair processes are engaged postflight, promoting muscle regeneration upon terrestrial return (80). This study supports previous work that showed elevated MCP-1 and IL-1RA expression postflight (180-d mission) (81), which may represent increased M2 populations postflight; however, more studies are necessary. In addition, elevated M1 predominance in in-flight astronauts may also suggest compensatory mechanisms to maintain immune homeostasis and may be a phenotype associated at a particular time point in immune response kinetics. Therefore, research in this area should expand in the next few decades because of the addition of tissue organoids and 3D cell culture platforms that can undergo autonomous in situ experimentation on orbit to enlighten knowledge about these important immunological states.

### Experiments in spaceflight microgravity: mammalian animal models

ISS and shuttle spaceflight missions involving nonhuman animal models play a crucial role in delineating the mammalian immune system in microgravity. Rodent models have been primarily used because of their translational phenotypes to humans and possibilities for genetic manipulation. Although various studies have determined a role for spaceflight-induced immune alterations, time points of sample collections (in-flight versus postflight return) have complicated interpretations. Generally, many reports suggest postflight immune dysfunction, with recovery to near baseline. For example, male 8- to 9-wk-old Sprague Dawley rats flown on Spacelab Life Sciences (SLS)-1 displayed significantly decreased counts of total WBCs, lymphocytes, and monocytes, with slightly increased neutrophils 3 d postflight of a 9-d mission (82). Similarly, on SLS-2 (14-d mission), 8- to 9-wk-old Sprague Dawley rats were analyzed postflight, displaying reduced T lymphocyte activity after mitogenic stimulation, which recovered by day 14 postflight (83). SLS-2 rats also displayed increased numbers of tingible body macrophages (germinal center, apoptotic cell engorged phagocytes) (84) within multiple lymphoid tissues, including thymic, spleen white pulp, and inguinal lymph nodes, at landing. These studies concluded elevated cellular apoptosis and macrophage phagocytosis in the spaceflight environment. However, the authors were unable to rule out whether microgravity- or re-entry–induced hypergravity caused these observations. In addition, no tingible bodies were observed at 9 d postflight, suggesting immune system readaptation upon return to Earth’s gravity (85).

Neuroendocrine cross-talk with the immune system in the spaceflight environment has also been characterized in rodent studies. For instance, 11-wk-old female *C57BL/6J* mice flew on the Space Shuttle Atlantis (STS-135) for 13 d, and tissues were collected within 3–5 h postlanding. The results displayed elevated ROS, increased gene expression of phagocytic activity (endosome and peroxisome formation), and elevated corticosterone levels in adrenals and livers, compared with ground controls (86). Because these analyses were performed at landing, readaptation to Earth’s gravity forces via mechanotransduction signaling pathways may have been involved in immune response activity.

In the earlier study (86) and in other missions, decreased thymus organ mass has been reported postflight (85, 87). In addition, chronic elevation of corticosterone levels can induce thymocyte apoptosis (88); therefore, elevated corticosterone observed in the spaceflight environment may disrupt thymic integrity. In line with this, physiological stress-inducing catecholamine and glucocorticoid production can regulate neutrophil demargination (neutrophil migration from vessel walls into blood circulation) processes (89) and may explain elevated neutrophil-to-lymphocyte ratio that is observed in blood in flight and at landing (32, 54, 90). However, additional studies that assess integrative physiological system cross-talk with immunity are necessary to fully comprehend immunological states induced by the spaceflight environment, along with postflight phases of recovery.

Furthermore, age-related thymic involution has also been associated with immunological processes (91). Accelerated cellular aging has also been described as a consequence of the spaceflight environment in humans (11, 12). Because thymic size is reduced in flight and in ground models of simulated microgravity (85–87), one study sought to determine whether this process could be reversed in the presence of gravity. To test this, 8- to 9-wk-old *C57BL/6J* males were exposed to 1 *g* artificial gravity centrifugation on board the ISS for 35 d, which showed partially rescued thymic involution was achieved as compared with on orbit mice without exposure to artificial gravity. They further determined artificial gravity mitigated impaired thymic cell-cycle-related gene expression, preserving thymic cell proliferation (92). These results indicate spaceflight may impair central immune tolerance processes, via lacked mechanotransduction mechanisms experienced in spaceflight microgravity; however, artificial gravity exposure diminished the rate of cellular turnover and further presents a unique countermeasure in flight that may partially reverse the effects of cellular aging.

Apart from aging studies, immune developmental studies in response to spaceflight exposure are rare. However, one study examined the impact of an 11-d shuttle mission in pregnant rodent dams (9-d gestation at launch), fetuses (unilateral hysterectomy 3 h postlanding), and pups (immediate postbirth euthanasia) immune development. The results showed little to no changes in immune parameters of developing offspring. However, reduced splenocyte blastogenesis and IFN-γ production (poststimulation) was observed in dams (93). Although no immune differences were identified in pup and fetal tissues, exposure to the spaceflight environment occurred at a developmental time frame (day 9) that yielded no effect. However, there is still a need to study earlier gestation time points exposure (before day 9) to the spaceflight environment, as well as in-flight gestation studies, which should be at the forefront in coming years.

As a measure of lymphocyte function and immune performance, tolerance mechanisms have been assessed in spaceflight. In one study, OVA Ag was autonomously administered via implanted minipumps into female 6- to 8-wk-old, OT-II adoptively transferred, CD45.1 congenic mice during a 15-d shuttle mission. Postflight lymphocyte stimulation assays showed spaceflight caused heightened inflammatory responses and reduced T cell tolerance via impaired T_regulatory_ cell generation and/or maintenance (55). These results suggest Ag processing/presentation pathways and/or MHC-TCR communications may be disrupted in spaceflight, further supporting spaceflight-induced immune dysfunction. In another study, 35-wk-old female *C57BL/6NTac* spleens were collected on board the ISS on day 21 postlaunch, which displayed subtle VDJ recombination sequence differences with both short-duration spaceflight and ground controls (94), indicating no significant differences in Ig diversity as a result of spaceflight. However, assessment of the Ab repertoire after longer duration, in particular on missions to the Moon or Mars, still remains an open question.

### Experiments in spaceflight microgravity: human studies

Majority of spaceflight immune studies originate from data gathered on missions to the Moon during the Apollo era or from flights in low-Earth orbit space shuttles, commercial shuttles, or the ISS. This perspective aims to capture studies that characterize the immunological states of the spaceflight environment as it relates to microgravity. However, there are other various environmental risks in the spaceflight environment that influence the immune response apart from microgravity, such as ionizing radiation or circadian disruption, for example. Thus, the immune response to spaceflight microgravity is complex and difficult to pinpoint. Nonetheless, collective features from various missions describe a central immune theme associated with the spaceflight microgravity environment, including immune exhaustion, senescence, and inflammaging.

For one, blood collected in-flight from male and female astronauts (45–53 y old) on various shuttle missions (10–15 d, short duration) display increased total WBC and granulocyte counts (95). In addition, virus-specific T cell function was impaired during and in postflight analysis, suggesting immune exhaustion and/or senescence, along with elevated TNF-α, IFN-α, and IFN-γ expression levels detected in plasma collected in-flight (95), indicating persistent inflammation. In another short-duration (10- to 13-d) mission, whole blood gene-expression profiling postflight compared with baseline controls showed gene pathways involved in oxidative stress and cellular repair processes. Downregulation of the antioxidant gene, glutathione peroxidase (GPX1), further indicated elevated ROS may trigger immune activity in the spaceflight environment (96). In line with this, other short-duration missions studied the effects of spaceflight on neutrophil (97) and monocyte (98) functions postflight. The findings revealed elevated neutrophil counts, no difference in monocyte counts, and impaired phagocytosis and oxidative burst reactions in both cell types. However, trends in elevated oxidative burst in both neutrophils and monocytes at landing compared with preflight controls were noted, supporting oxidative stress mechanisms are dysregulated in the spaceflight environment (97, 98). Indeed, elevated mitochondrial dysfunction, which increases oxidative stress–induced damage and inflammation, was recently reported in 4- to 6-mo mission astronaut blood (99) and is also a feature of immune senescence (15, 17, 18). Further, oxidative stress-induced DNA damage in PBMCs was reported in up to 1-y mission astronauts [*n* = 3 (12) and 11 (11)], displaying significantly shorter telomeres, lower telomerase activity, and increased chromosomal inversion frequencies than age- and sex-matched ground controls that persisted up to 6 mo postflight return (11, 12). These results further support accelerated aging in the spaceflight environment.

In addition, a large cohort of ISS astronauts (*n* = 59; 47 males and 12 females) displayed elevated markers of inflammation in plasma (4- to 6-mo mission duration), including IL-1α, IL-1β, and IL-RA, with continuous IL-1RA expression detected immediately after flight return (81, 100). Importantly, a separate report observed inflammaging shifts induced during long-duration flight (>140 d) in cosmonauts, including elevated neutrophil activity, amplified TNF and IL-1β response to fungal Ags, reduced anti-inflammatory mechanisms, and elevated CD8^+^ memory T cell production that persists up to 30 d postflight (54). Therefore, mild, persistent inflammation is induced after exposure to the spaceflight environment and is also a notable feature of immune senescence and immunological aging (20, 21).

To prepare for deep space exploration missions, current studies primarily focus on the effects of long-duration missions (≥6 mo) on immunological states. For instance, one mission (6 mo, average 53 y old) displayed elevated total WBCs, granulocytes, and NK cell counts, whereas postflight stimulation of lymphocytes displayed reduced activation (CD69^+^) in both CD4^+^ and CD8^+^ T cell subsets (90). In another study, NK cell function was impaired by 3 mo in flight (39–52 y old, 6-mo mission), which was more pronounced in first-time mission astronauts, compared with multimission astronauts (101). Being important cells involved in immune surveillance and response to viral infections (102), and because astronauts experience latent viral reactivation of herpes virus and EBV shedding in flight (103–105), targeting NK cell activity in the spaceflight environment may be a strong candidate for countermeasure design, and therefore warranting further studies on these cell types.

More than 550 humans have flown in space, many of whom have spent >300 d throughout their spaceflight careers. Yet, only a few have spent >300 d in space on a single mission. One historical mission lasting for 340 d, commonly known as the NASA Twins Study, involved two genetically identical twin astronauts (51 y old). This study was designed to assess the effects of long-duration spaceflight (*n* = 1) compared with ground (*n* = 1) (106) in two genetically identical individuals who had similar career paths and life activities. In this study, dynamic inflammatory signatures were measured throughout the mission. Collectively, multiple cytokine levels were elevated in spaceflight and remained elevated throughout flight, whereas immediately upon landing, cytokine levels increasingly spiked and then returned to baseline around a month later. Although only a sample size of *n* = 1 (flight versus ground), this study provides evidence of immune activity profiles to consider on future long-duration spaceflight missions.

### Current limitations of simulated and space microgravity experiments

Although multiple studies have expanded our knowledge on the immunological states in microgravity, which can better prepare astronauts for future low-Earth orbit and deep space missions, there are still some shortcomings. Modeling the spaceflight microgravity environment on Earth is challenging. For instance, reduced mobility and unloading experienced during HDTBR (and HU) versus the lack of gravity forces experienced during spaceflight microgravity are not similar models. Yet, fluid shifting–caused vascular damage and mechanotransductive activity may produce similar biological consequences, warranting the use of these models. In addition, because complex cellular interactions and dynamics of immunity exist, there will also be interpretative disparities across research. For instance, the immune response generated after day 1 in microgravity is not the same response on day 180. Therefore, longitudinal immune performance should be carefully evaluated to better identify suitable countermeasure intervention time points. Also, personalized immune responses add a layer of complexity and require careful consideration for countermeasure targeting and administration. In addition, there are considerable gaps in knowledge regarding comparable durations of exposure in ground modeling that mimic flight, also requiring further study. For example, although relevant for immune characterization, immune responses in mice during a 30-d HU study versus in humans during a 5-d HDTBR study may not produce similar biological outcomes as compared with astronauts on long-duration missions to the Moon or Mars. Therefore, carefully planned experimental timelines and exposure types that can model various future spaceflight mission timelines are important for future studies.

On orbit, sample collection methods and analysis are limited because of cellular viability and storage requirements and the nature of fluids in microgravity. In addition, human studies typically analyze easily accessible biofluid (i.e., blood, saliva, and urine) samples. In contrast, analysis human lymphoid tissues (i.e., thymus, spleen, or bone marrow) studies are rare; therefore, the use of rodent models in spaceflight research is necessary to better understand developmental immune organs and their performances in space and time. Other limitations for spaceflight experiments are typical delays in return of biological samples collected in flight, as well as the sample sizes that can be collected in flight, which can both confound results. Also, landing stresses experienced, such as in hypergravity, vibration, and heat exposures, can cause sample viability issues and provoke immune responses, confounding results collected from the microgravity environment, and should be carefully characterized through metadata analyses. However, with the advent of an onboard flow cytometer (107), real-time imaging platforms, and other autonomous payload developments, current experimental constraints will evolve, producing more reliable in-flight measurements and results. In addition, 3D tissue engineering culture systems have greatly increased in popularity but still remain underused (10, 108, 109). However, these tools will provide an exceptional alternative to assess immune cell migration patterns, interactions, and activity in both microgravity and terrestrial environments. Furthermore, open science data repositories and sample sharing programs have greatly enhanced scientific rigor, sample sizes, and accessibility. Therefore, as more tools, instruments, and methods are improved, definitive results about immunological consequences of the microgravity environment can be concluded.

As sights are set toward deep space exploration, including missions to the Moon and Mars, it is important to prioritize the human experience. Spaceflight data collected over the last three decades have revolutionized our comprehension of immunological states in the microgravity environment. Furthermore, commercial spaceflight programs are also progressing and are contributing to the advancement of human research in the unique environment of spaceflight. Aside from microgravity, immunological responses to landing on Earth, the Moon, and Mars with respect to gravity and atmosphere will differ. Yet, very few studies have looked into immunological readaptation processes in different gravity environments, which is an important avenue for future research. Further, it has been demonstrated that artificial gravity does partially reverse impaired immune effects produced from microgravity exposure. Thus, it can be hypothesized that partial gravity may encourage partial immune homeostasis, yet this remains to be discovered.

In summary, as described through multiple studies, this perspective identified key immunological states influenced by the microgravity environment, including accelerated immunological aging phenotypes that closely resemble immune exhaustion and/or senescence (43, 45–47, 55, 73, 83, 93, 95, 99) and inflammaging (54, 55, 72, 74, 75, 95, 96, 99, 100, 106) (summarized in Fig. 2 and Supplemental Tables I and II). Proposed countermeasures, such as probiotics and artificial gravity to mitigate immune disruption in the spaceflight microgravity environment are also described. Therefore, maximizing research that reverses or mitigates these immunological states may be essential directives for future exploration missions.

## Supplementary Material

Supplemental Tables 1 (PDF)
