# Supplementary material for: The Impact of Microgravity on Immunological States
Source: Immunohorizons. 2023 Oct 19;7(10):670–82. doi: 10.4049/immunohorizons.2200063 (PMC10615652; doi:10.4049/immunohorizons.2200063)
Supplement: Supplemental Tables 1 (PDF) [file IH_2200063_Supplemental_1.pdf]

Supplemental Table 1. Summary table of immune studies in simulated microgravity environments.

Microgravity Model	Rodent (age/strain/sex)	Human (age, sex)	Metadata	Primary findings (compared to controls)	Reference
**RCCS**		Whole Blood	20rpm, 12h	↑ROS & MPO	Paul et al., 2020a
		21-55y/o, M/F	5rpm, 18h	↓CD4, CD8, NK function; ↑T_{reg} responses	Spatz et al., 2021
		U937 Cell line	72h	↑ IL-1α/β, IL-8, MCP-1, M-CSF, MIP-1α, MIP-1β, & TGF-β1	Maier et al., 2006
		Lymphocytes	10rpm, 72h-20D	↓IgM, IgG & lymphocyte proliferation	Fitzgerald et al., 2009
	Humanized mice (human RCCS-exposed lymphocytes)		10rpm, 12h	↑Tumor burden & defective anti-tumor lymphocytes	Mylabathula et al., 2022
	6 to 8-week-old, *C57BL/6*, F		15rpm, 24-168h	↓Mast cell degranulation	Kim et al., 2022
	6 to 8-week-old, *BALB/c*, F		6rpm, 48h	↓IL-1β, IL-2, IL-3, TNF-α, IFN-γ, & splenocyte proliferation	Hales et al., 2002
	RAW264.7 Cell line (M0, M1, & M2 differentiated)		14rpm, 72h	↓TNF-α; ↑IL-12, IL-10, and VEGF; ↑IL-6 (M1 only)	Ludka et al., 2021
	JAWS II DC Cell line		16rpm, 3D	↑pSTAT-5, pERK1/2, and p-mTOR, MHC-I and -II, CD80/86	Tackett et al., 2019
	JAWS II DC Cell line		16rpm, 4-14D	↓maturation markers and T cell immunogenicity	Tackett et al., 2019
	OT-II T Cell line + JAWS II DC Cell line + Ovalbumin		14rpm, 24h (co-culture) 48hr T cell culture	↑IL-2 and immune synapse formation	Bradley et al., 2017
	OT-II T Cell line + JAWS II DC Cell line + Ovalbumin		14rpm, 24h (co-culture) 120hr T cell culture	↓IL-2<br>↑CTLA-4	Bradley et al., 2017
**2D CLINOSTAT**	NR8383 Rat Cell line		60rpm, 20min	↓ROS	Adrian et al., 2013
		CD4+ T cell	60rpm, 5 & 60min	↓CD3, ZAP70 (5-min)<br>↓IL-2R (60min)	Tauber et al., 2015
		PBMC (anti-CD3 stimulated)	30rpm, 24h	↓IL-2R & CD69<br>↓cell proliferation	Hashemi et al., 1999
		CD4+ T cell (anti-CD3+CD28 stimulated)	14rpm, up to 90-min	↓IκBα, pZAP, pζ, pSLP<br>↑PLC<br>ND pELK, pRSK, pERK	Simons et al., 2010
**HU**	6 to 8-week-old, *ICR*, F		24h	↑WBC, neutrophils, lymphocytes, monocytes, and eosinophil counts compared to PWS	Wilson et al., 2012
	5 to 6-week-old, *ICR*, F		24h	↑Acute phase reactant proteins and soluble CD14	Zhou et al., 2012
	5 to 6-week-old, *ICR*, *BALB/c*, & *C3H/NeN*, M/F		10D	↓Clearance of bacteria, neutrophil mobilization; ↑corticosterone level in HU	Li et al., 2014
	8 to 10-week-old, *C57BL/6*, F		14D	↑Bacterial species, neutrophilia, and IL-1β in HU with DSS	Li et al., 2015
	16-week-old, *C57BL/6NJ*, F		14- & 30-D	↑Neutrophils & NLR	Paul et al., 2020a
	8 to 10-week-old, *C57BL/6*, F		28D	↑*Clostridium* species and ↓colonic goblet cell number	Shi et al., 2017
**PWS**	6 to 8-week-old, *ICR*, F		24h	↓WBC, neutrophils, lymphocytes, monocytes, and eosinophils compared to HU	Wilson et al., 2012
**HDTBR**		31-33y/o, M	3D	↓T cells & CD62L on granulocytes; ↑sCD62L shedding	Feuerecker et al., 2013
		26-38y/o, M	21D	↓IL-2, IFN-γ, TNF-α, and IL-10	Kelsen et al., 2012
		23-38y/o, M	65- & 105-D	↑ *β*2-integrin on neutrophils, IL-6, NK cell, & cortisol (150D); ↓CD4/CD8 ratio	Choukèr et al., 2001

"D" denotes days, "M" denotes months

Supplemental Table 2. Summary table of immune studies in spaceflight microgravity environments.

Microgravity Model	Rodent (age/strain/sex)	Human (age, sex)	Metadata	Primary findings (compared to controls)	Reference
**SPACEFLIGHT**	8 to 9-week-old, Sprague-Dawley, M		9D (SLS-1)	↓WBC, lymphocytes, & monocytes; ↑slight neutrophils 3D post-flight	Allebban et al., 1994
	Rat (Taconic Biosciences), F		11D	↓Blastogenesis of spleen cells & IFN-γ in dams post-flight	Sonnenfeld et al., 1998
	11-week-old, *C57BL/6J*, F		13D (STS-135)	↑ROS, phagocytic activity, corticosterone; ↓thymus mass post-flight	Pecaut et al., 2017
	8 to 9-week-old, Sprague-Dawley, M		14D (SLS-2)	↓T cell activity; ↑ tingible body macrophages post-flight; ↓thymus mass post-flight	Lesnyak et al., 1996<br>Gotur et al., 2020<br>Congdon et al., 1996
	6 to 8-week-old, CD45.1 congenic OT-II, F		15D	↑Inflammation; ↓T cell tolerance and T_{regs} post-flight	Chang et al., 2015
	35-week-old, *C57BL/6NTac*, F		21D	ND in antibody diversity	Ward et al., 2018
	8 to 9-week-old, *C57BL/6J*, M		35D	↑Thymic involution compared to 1g controls post-flight	Horie et al., 2019
	B6MP102 Macrophage Cell line		STS-37 & STS-43	↑IL-1 & TNF-α in flight	Chapes et al., 1992<br>Chapes et al., 1994
	Splenic lymphocytes		STS-37	↑IFN-α in flight	Chapes et al., 1992<br>Chapes et al., 1994
	Lymph node cells	PBMC	STS-43	↑IFN-γ in flight	Chapes et al., 1992<br>Chapes et al., 1994
		Bone marrow (Lin- cells) w/ M-CSF & IL-3/IL-6 stimulation	12D	↓Macrophage differentiation/polarization	Shi et al., 2021
		Macrophages (M1)	11- & 30-D	↑Number; ↓ICAM-1 (11-day)	Tauber et al., 2017
		Avg. 44y/o (5D); 41y/o (9-11D); 77y/o (n=1), M/F	5-11D	↑Neutrophil counts, oxidative burst compared to pre-flight; ↓phagocytosis post-flight	Kaur et al., 2004 and 2005
		45-53y/o, M/F	10-15D	↑TNF-α, IFN-α, and IFN-γ, WBC and granulocytes; ↓T and NK cells & virus-specific T cell activity	Crucian et al., 2013
		38-47y/o, M/F	10-13D	↑Oxidative stress and cell repair	Barrila et al., 2016
		Avg. 46y/o, M	Approx. 4M	↑Neutrophil activity, TNF, IL-1β, CD8+ T cell post-flight	Buchheim et al., 2019
		Avg. 47y/o, M/F	4-6M	↑Mitochondrial dysfunction, oxidative stress, & inflammation	da Silveira et al., 2020
		Avg. 44y/o (ISS) & 36y/o (ground), M/F	6M	↓NK function (3M) post-flight	Björkström et al., 2022
		53y/o, M/F	Approx. 6M	↑WBC, granulocytes, NK count; ↓T cell activation ($CD4^+$ & $CD8^+$ subsets) post-flight	Crucian et al., 2015
		51y/o, M	Approx. 11M	↑Inflammatory response post-flight (n=1)	Garrett-Bakelman et al., 2019
		47.0 ± 5.6 y/o, M/F	Approx. 6M	↑IL1-RA post-flight	Crucian et al., 2014<br>Paul et al., 2020b
		35-55y/o, M/F	Approx. 6M & 11M	↓Telomere length post-flight	Luxton et al., 2020a<br>Luxton et al., 2020b

“D” denotes days, “M” denotes months
